# Optimal Routing for Time-Driven EH-WSN under Regular Energy Sources

**DOI:** 10.3390/s18114072

**Published:** 2018-11-21

**Authors:** Sebastià Galmés

**Affiliations:** Department of Mathematics and Computer Science, University of Balearic Islands, 07122 Palma de Mallorca, Spain; sebastia.galmes@uib.es; Tel.: +34-669-130-240

**Keywords:** energy-harvesting wireless sensor network, solar radiation, energy consumption model, duty cycle, throughput, medium access control layer, routing, minimum hop count, shortest-path routing

## Abstract

The recent provision of energy-harvesting capabilities to wireless sensor networks (WSN) has entailed the redefinition of design objectives. Specifically, the traditional goal of maximizing network lifetime has been replaced by optimizing network performance, namely delay and throughput. The present paper contributes to this reformulation by considering the routing problem for the class of time-driven energy-harvesting WSN (EH-WSN) under regular or quasi-periodic energy sources. In particular, this paper shows that the minimum hop count (MHC) criterion maximizes the average duty cycle that can be sustained by nodes in this type of scenarios. This is a primary objective in EH-WSN, since large duty cycles lead to enhanced performance. Based on a previous result, a general expression is first obtained that gives mathematical form to the relationship between duty cycle and traffic load for any node in a time-driven EH-WSN fed by a regular energy source. This expression reveals that the duty cycle achievable by a node decreases as its traffic load increases. Then, it is shown that MHC minimizes the average traffic load over the network, and thus it maximizes the average duty cycle of nodes. This result is numerically validated via simulation by comparison with other well-known routing strategies. Accordingly, this paper suggests assigning top priority to the MHC criterion in the development of routing protocols for time-driven EH-WSN under regular energy sources.

## 1. Introduction

Recent advances in wireless sensor networks have led to the development of energy-harvesting capabilities, which are expected to enable very long or even perpetual operation. In parallel, the design focus has progressively been shifted from maximizing network lifetime, usually defined as the time until first node death, towards optimizing network performance, basically delay and throughput [1]. Therefore, despite energy issues in energy-harvesting wireless sensor network (EH-WSN) cannot be disregarded, due to the time-varying nature of ambient energy sources, the new design priorities demand for revising current protocols for battery-powered WSN. Note that such new priorities represent a change in statistical sense too: While the design goal in battery-powered WSN is to maximize a lower bound (time until first node death), the goal in EH-WSN is to maximize an average (average performance). Such a difference obviously conditions the design of protocols.

Most contributions on battery-powered WSN have focused on the Medium Access Control (MAC) layer, as this plays a fundamental role in the energy expended by a sensor node. Research activity on EH-WSN is also giving priority to the development of MAC protocols, but relaxing the constraints on energy consumption. Specifically, the new objective at the MAC layer is to increase the duty cycle of nodes as much as possible according to their individual energy harvesting patterns, as opposed to the common system-wide reduced duty cycle of battery-powered WSN. References [2,3] provide, respectively, comprehensive surveys on MAC protocols for battery-powered and energy-harvesting WSN.

The network layer is also under review. In fact, a few routing protocols for EH-WSN have been recently proposed with the aim of optimizing performance metrics, such as delay and throughput, again in contrast to the network lifetime maximization pursued by traditional routing protocols [4,5]. One of the contributions is [6], which proposes an algorithm rather than a protocol. This algorithm, known as Energy-opportunistic Weighted Minimum Energy (E-WME), calculates the cost of each node as an exponential function of its residual energy, and then uses shortest-path routing on the basis of this metric. In addition, it offers high versatility, as it can be easily incorporated into different routing implementation schemes, like proactive or on-demand. In [7], a modification on the well-known Collection Tree Protocol (CTP) is proposed, namely Loop-aware CTP (La-CTP). This protocol introduces several mechanisms to effectively suppress the occurrence of loops and unlock unavoidable loops in moving packets across an EH-WSN. In general, these loops arise from temporary departures of nodes that stop working to enter a recharging state. In [8], a hierarchical topology is assumed and a centralized routing algorithm containing two parts is proposed. The first part consists of a genetic-based unequal clustering algorithm, which is run by the base station to form clusters of unequal size. Here, information about location, energy level and energy harvesting rate of all nodes is used. After clustering, the base station executes another algorithm to construct an inter-cluster routing among all cluster heads. This algorithm also takes into account the energy harvesting condition of the participant nodes, in this case the cluster heads. Another contribution is [9], which proposes the Energy Harvesting Opportunistic Routing (EHOR) protocol for multi-hop EH-WSN. This kind of opportunistic protocol uses a region-based approach to determine the optimal forwarders for every packet, by taking into account the energy condition of all nodes, and the fact that some of them are in recharging state and thus they are temporally unavailable. In [9], a linear topology is assumed. Then, EHOR is extended to 2D topologies in [10], under the name of Adaptive Opportunistic Routing (AOR). Finally, [11] and [12] propose modifications to the Low-Energy Adaptive Clustering Hierarchy (LEACH) protocol [13], which is the most well-known hierarchical protocol for battery-powered sensor networks. Particularly, an Energy Potential (EP) function is introduced in [11] to quantify the node capability for harvesting energy from the environment. The resulting protocol, called EP-LEACH, uses the EP function in the cluster head selection strategy. Compared to LEACH, it is shown to exhibit better performance in terms of lifetime and throughput. A similar approach is followed in [12], but in this case a solar energy prediction model based on a neural network is used to estimate the energy that is to be harvested by a node in a short term horizon. This estimate and the node current residual energy are then combined to determine the probability of the node to become a cluster head. Again, simulation results demonstrate that the proposed clustering method outperforms that of traditional LEACH with respect to average residual energy of nodes and network throughput.

A common characteristic of the above routing protocols (except La-CTP) is that they assume an oversimplified MAC layer, where the only sources of energy consumption are the pure transmission and reception of packets. Whereas this is true for MAC protocols based on Time Division Multiple Access (TDMA), it is far from reality for the rest of them, since they typically involve other sources of energy expenditure, namely idle listening, packet retransmissions, etc. Another drawback of current routing protocols for EH-WSN is their reliance on heuristic algorithms, which do not necessarily lead to optimal performance and/or incur significant overhead in terms of computational time or control traffic. Whereas these approaches may constitute the only viable methodology for EH-WSN subject to unpredictable traffic loads and/or energy sources, the regular patterns exhibited by some energy-harvesting processes, like those based on solar power, and some traffic loads, like those resulting from periodic monitoring applications, suggest the possibility of analytical treatment.

This paper focuses on EH-WSN devoted to periodic monitoring, also known as time-driven EH-WSN. Under the time-driven paradigm, nodes periodically sense the environment and report the corresponding data to the base station, usually via multi-hop communication. This is the case of numerous sensor-based applications, which span a great diversity of monitored variables. Typically, the design of the MAC layer is based on duty-cycling the communication activity of nodes, in order to avoid fast depletion of energy resources, due to idle listening. Since the reporting frequencies imposed by monitoring applications are commonly very low, every reporting period may contain thousands of unused duty cycles, due to the relatively small number of packets being transmitted or forwarded. Therefore, in spite of having duty-cycled the communication activity, idle listening is still a dominant component in the energy wasted by time-driven sensor networks. Note that this is true for both, battery-powered and energy-harvesting sensor networks, though it is expected that the latter allows for larger duty cycles, or for larger network sizes (in terms of the number of nodes) under the same duty cycle.

Particularly, the present work follows an analytical approach to address the routing problem in time-driven (duty-cycled) EH-WSN operating under regular or quasi-periodic energy sources, among which solar radiation is the most representative example. Such approaches rely on a comprehensive energy consumption model for the MAC layer, which takes into account all sources of energy consumption, and on the application of the so-called energy neutral condition for EH-WSN. The following assumptions are adopted:

- Both planar and hierarchical topologies are taken into account. In the latter case, the routing problem focuses on interconnecting the cluster heads to the base station.
- At most, data aggregation is considered at the intra-cluster level in the case of hierarchical topologies. This is consistent with the trend of deploying sensor networks over larger and larger areas, a fact that reduces correlation among data from different sub-regions.
- Homogeneous distribution of the traffic workload generated by nodes (offered traffic). This means that all sensor nodes generate the same amount of packets per unit of time.
- The transmit power of nodes is set to the maximum, that is, power control is disabled. This implies that the energy wasted by a node to transmit a packet does not depend on distance. Accordingly, the distance between a node and its receiver is not relevant, except for the fact that it must be lower than the transmission range.

On the basis of these assumptions, this paper demonstrates that the MHC criterion should be prioritized in the design of routing strategies and protocols for time-driven EH-WSN under regular energy sources. More specifically, the detailed contributions of this paper can be outlined as follows:

- Based on a previous result regarding the energy consumed by TinyOS sensor nodes [14], a comprehensive model is derived to characterize the energy consumption of nodes in generic time-driven duty-cycled wireless sensor networks.
- general formulation is then obtained which relates duty cycle and traffic load for time-driven duty-cycled EH-WSN.
- It is mathematically shown that, in addition to the obvious minimization of path delay, the minimum hop count criterion also minimizes the average traffic load over the network, and thus it maximizes the average duty cycle of nodes. In turn, this contributes to minimizing the link-level delay and maximizing the average network throughput that can be sustained by the whole network.

The rest of this paper is organized as follows. In Section 2, from the result obtained in [14], a generic energy consumption model for time-driven duty-cycled sensor networks is developed. Though the analysis performed in this paper is valid for any regular or quasi-periodic energy source, special attention is dedicated to solar radiation, as it is the most representative example. Accordingly, a review of solar-based energy-harvesting models is provided in Section 3. By assuming a regular or quasi-periodic energy source, the condition for energy neutral operation is formulated in Section 4. In Section 5, it is shown that the MHC metric minimizes the average traffic load over the network and maximizes the average duty cycle of nodes. In Section 6, numerical results are obtained and compared with those from other routing strategies. Finally, Section 7 concludes the paper with suggestions for further research.

## 2. Energy Consumption Model

There are two types of duty-cycled MAC protocols in sensor networks, namely synchronous and asynchronous. Synchronous protocols are based on TDMA and thus represent an extreme case of duty-cycling, since nodes are only active during the specific timeslots devoted to transmit or receive. This results from the fact that any transmitter and its receiver wake up at the same time. However, such protocols require tight synchronization among nodes, and exhibit significant limitations in terms of scalability and adaptiveness to changing traffic conditions. These disadvantages make asynchronous protocols more attractive, at the expense of more energy consumption and some throughput degradation.

**Figure 1:**
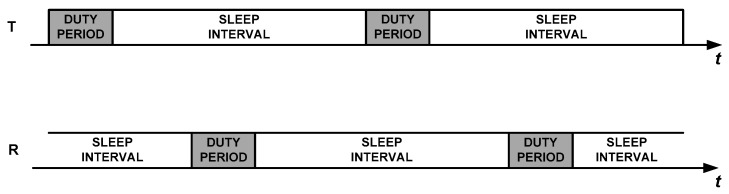
Uncoupled duty cycles between a transmitter node (T) and a receiver node (R) in asynchronous communication.

In asynchronous communication, nodes have their duty cycles completely decoupled, as it is shown in Figure 1. Thus, two general mechanisms have been proposed in the literature in order to link a transmitter that has data to send with its receiver: Low Power Listening (LPL) and Low Power Probing (LPP). In LPL, the responsibility of the task is shifted to the transmitter, which uses a duty period to initially send a long preamble or a burst of advertisement packets in order to warn the receiver that it has pending data. It can also send a repetitive sequence of the data packet itself. Upon waking up and detecting the preliminary signalling or the sequence of data packets, the receiver stays awake until the transmission process is completed, meaning that a full data packet has been correctly received. Examples of implementations of LPL are X-MAC [15], Aloha with preamble sampling [16], B-MAC [17], and BoX-MAC-1 and BoX-MAC-2 [18]. In contrast, in LPP [19], it is the receiving node that periodically sends small packets called beacons or probes, to announce that it is awake and ready to receive data. A node willing to send a packet turns its radio on and waits for a probe. Upon receiving a probe from the intended destination, it sends an acknowledgment and, subsequently, the data packet. The most representative LPP protocols are RI-MAC [20] and A-MAC [21].

As stated above, both synchronous and asynchronous mechanisms are part of the class of duty-cycled MAC protocols, as in both cases nodes use non-activity periods to switch to sleep mode in order to save energy. However, usually the term duty-cycled MAC protocol refers to the asynchronous version, which is the most extended implementation [22]. This is the focus of the present paper.

### 2.1. Energy Consumption Model for the LPL mechanism in TinyOS Sensor Nodes

**Figure 2:**
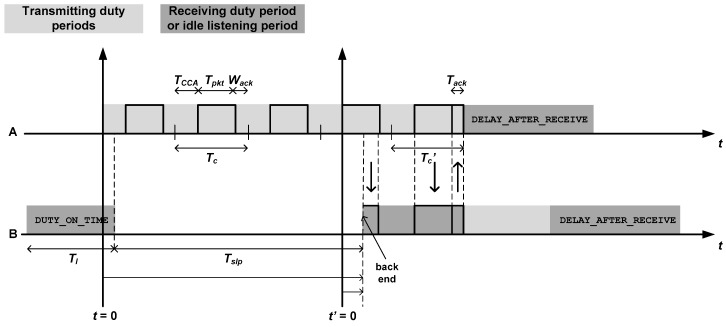
Operation of Low Power Listening (LPL) in TinyOS sensor nodes: Node A transmits a packet to node B, which receives and forwards this packet.

For the sake of completeness, this subsection recalls the main results obtained in [14] about the energy consumed by the LPL mechanism implemented in TinyOS sensor nodes. Figure 2 describes this mechanism, where node A transmits a packet to node B, which receives and forwards such packet to the next hop (not shown). As it can be noticed, node A sends the packet repetitively until node B wakes up, captures the full packet and sends back an acknowledgment packet. The figure introduces the following temporal magnitudes:

- $T_{CCA}$: Time for clear channel assessment. This is the time required by the sending node to check that there are no ongoing transmissions on the channel.
- $T_{pkt}$: Packet duration.
- $W_{ack}$: Waiting time for the acknowledgment from the receiver. If no acknowledgment is received, the transmission was unsuccessful and the transmitter tries again.
- $T_{ack}$: Duration of acknowledgment packets.
- $T_{c}=T_{CCA}+T_{pkt}+W_{ack}$.
- $T_{c}^{\prime }=T_{CCA}+T_{pkt}+T_{ack}$.
- $T_{l}$: Nominal duration of duty periods, also known as DUTY_ON_TIME in TinyOS nomenclature. This is, in fact, the duration of duty periods in absence of traffic activity (minimum duration).
- $T_{slp}$: Duration of sleep periods. If DC denotes the nominal duty cycle (in percentage), we can set up the following equality: $DC=\frac{T_{l}}{T_{l}+T_{slp}}100$.
- DAR: This is the DELAY_AFTER_RECEIVE, a period of time that a node remains active after completing a traffic task, either a transmission (node A) or a reception (with subsequent forwarding) (node B). Note that the name of this magnitude does not reflect its full role, as it suggests that it only takes place after a packet reception.

According to the assumption that the offered traffic is homogeneously distributed over the network (Section 1), let us assume, with no loss of generality, that each node reports one packet per communication round. Therefore, if a given node X has σ(X) descendants in the routing tree, its traffic load is precisely σ(X), since this node has to receive and forward σ(X) packets (aside from transmitting its own packet). In [14], an accurate expression is provided for the average energy consumed by a TinyOS sensor node in every communication round of a time-driven application:

(1) $$\varepsilon \left[E_{round}\left(X\right)\right]=\sigma \left(X\right)\cdot \varepsilon \left[E_{R}\left(X\right)\right]+\left(\sigma \left(X\right)+1\right)\cdot \varepsilon \left[E_{T}\left(X\right)\right]+\left(\frac{T_{rnd}}{T_{l}+T_{slp}}-\left(\sigma \left(X\right)+1\right)\right)\cdot E_{l}.$$

In the above equation, $E_{R}\left(X\right)$ denotes the energy wasted to receive a packet, $E_{T}\left(X\right)$ is the energy wasted to transmit a packet, $T_{rnd}$ is the duration of a communication round and $E_{l}$ is the energy consumed in idle listening by every duty period without traffic activity. ε[·] is the expectation operator. The presence of this operator is due to the random asynchrony between the duty periods of the two communicating nodes (see Figure 2). This randomness is reflected in two components: The number of tries performed by the transmitter until it receives an acknowledgment (node A in Figure 2), and the fraction of receiver duty period until the start of a full packet (node B in Figure 2). Specifically, the two expectations in Equation (1) can be formulated as follows:

(2) $$\varepsilon \left[E_{T}\left(X\right)\right]=\left(\varepsilon \left[k\right]-1\right)\cdot E_{c}\left(X\right)+E_{c}^{\prime }\left(X\right)+E_{l}^{DAR},$$

(3) $$\varepsilon \left[E_{R}\left(X\right)\right]=\varepsilon \left[E_{fd}\right]+E_{rx}^{pkt}+E_{tx}^{ack}.$$

In these equations, ε[k] and $\varepsilon \left[E_{fd}\right]$ are, respectively, the expected number of tries and the expected duration of a fragment of duty period, $E_{c}\left(X\right)$ is the energy wasted in non-successful transmission cycles, whose duration is $T_{c}$, and $E_{c}^{\prime }\left(X\right)$ is the energy wasted in a successful transmission cycle, the duration of which is $T_{c}^{\prime }$ (Figure 2). Additionally, $E_{l}^{DAR}$, $E_{rx}^{pkt}$ and $E_{tx}^{ack}$ are, respectively, the energy consumed in a DELAY_AFTER_RECEIVE period, the energy wasted to receive a packet and the energy wasted to transmit an acknowledgment. Note that, implicitly, it has been assumed that power control is disabled, because the energy wasted to transmit an acknowledgment, which is part of the energy wasted to receive a packet, does not exhibit any dependence on the specific node to which the acknowledgment is sent. This is reflected in the fact that the traffic load generated by all descendants of node X has been grouped into a single term σ(X) in Equation (1). Whereas this contributes to simplifying the analysis it does not cause any detriment on the generality of subsequent results.

### 2.2. Approximate Energy Consumption Model

In this subsection, the model just described is generalized to any implementation of LPL or LPP. To start with, Equation (1) can be simplified by removing several terms that are very specific to the implementation of LPL in TinyOS and do not have a significant contribution. Accordingly, the following approximate energy consumption model can be derived:

(4) $$\varepsilon \left[E_{round}\left(X\right)\right]≅\sigma \left(X\right)\cdot E_{rxpkt},\;+\left(\sigma \left(X\right)+1\right)\left(\varepsilon \left[k\right]\cdot E_{txpkt}+E_{l}^{DAR}\right)+E_{l}\frac{T_{rnd}}{T_{l}}\frac{DC\left(X\right)}{100}.$$

In this equation, it has also been assumed that the number of duty cycles per communication round is very large compared to the amount of such duty cycles that are entailed to transmit or receive. This assumption is in agreement with the large reporting periods that typically characterize time-driven applications, though again it does not compromise the generality of the main results of this paper. The term DC(X) denotes the duty cycle of node X (expressed in percentage). On the other hand, the term ε[k] depends on the duty cycle of the parent node of node X, namely DC(p(X)). In effect, as it can be noticed from Figure 2, it is the dynamics of node B that determines the number of tries required by node A. The specific relationship between ε[k] and DC(p(X)) for TinyOS nodes can be found in [14], but it has been omitted here as it is not relevant to the analysis that follows.

**Figure 3:**
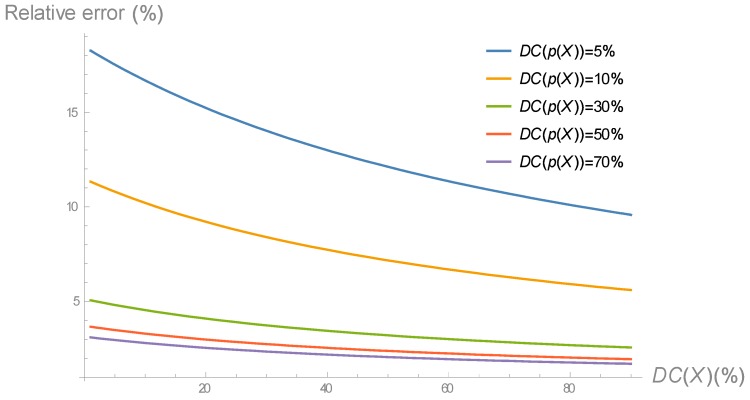
Relative error between exact and approximate energy consumption models for TinyOS sensor nodes.

Figure 3 shows the relative error between the exact model, given by Equation (1), and the approximate model is given by Equation (4), in terms of DC(X), for different values of DC(p(X). The rest of the parameters are given in Table 1. As it can be noticed, the relative error decreases as both duty cycles increase. Particularly, for duty cycles equal to or larger than 40%, the relative error is around 2%. As stated in Section 1, large (and heterogeneous) duty cycles are very common in EH-WSN, and thus the approximate energy consumption model can replace the exact one for TinyOS EH-WSN.

To proceed with the generalization, we can start by reformulating Equation (4) as follows:

(5) $$\varepsilon \left[E_{round}\left(X\right)\right]≅\sigma \left(X\right)\cdot E_{rxpkt}+\left(\sigma \left(X\right)+1\right)\cdot E_{txpkt},\;+E_{l}\frac{T_{rnd}}{T_{l}}\frac{DC\left(X\right)}{100}+\left(\sigma \left(X\right)+1\right)\cdot E_{trigger}\left(X\right).$$

Here, $E_{trigger}\left(X\right)$ represents the energy wasted by the transmitter (node X) to trigger the communication with its receiver (node p(X)). For TinyOS nodes, it can be expressed as follows, where ε[n] denotes the expected number of (unsuccessful) transmission tries before a correct data packet is received:

(6) $$E_{trigger}\left(X\right)=\varepsilon \left[n\right]E_{txpkt}+E_{l}^{DAR}.$$

A detailed analysis of Equation (5) reveals that all terms except the last one characterize the main sources of energy consumption in any time-driven duty-cycled sensor network, regardless of the particular platform. At the same time, specific implementation details about the triggering method can be assumed to be embedded into the variable $E_{trigger}\left(X\right)$. Accordingly, Equation (5) becomes appropriate to model a large variety of LPL and even LPP-based MAC protocols, and hence it can be used to formulate the condition for energy neutral operation in the next section. For the sake of completeness, we can attempt to infer a more explicit but still general formulation for $E_{trigger}\left(X\right)$. In the case of LPL-based MAC protocols, the following expression can be postulated:

(7) $$E_{trigger}\left(X\right)=\{\begin{array}{c} \varepsilon \left[n\right]E_{txpkt}+E_{extra},\;\;case\;1\\ \varepsilon \left[n\right]E_{txadv}+E_{extra},\;\;case\;2\\ \varepsilon \left[E_{fp}\right]+E_{extra},\;\;\;\;\;\;\;\;\;case\;3 \end{array}.$$

Here, cases 1, 2 and 3 correspond, respectively, to using a repetitive sequence of the data packet (LPL in TinyOS), a repetitive sequence of an advertisement packet or a long preamble. Moreover, ε[n] denotes the expected number of times that the data packet or the advertisement packet is transmitted before the data packet is fully received, $\varepsilon \left[E_{fp}\right]$ is the expected value of energy wasted in transmitting a fragment of preamble and $E_{extra}$ stands for any extra fixed-component of energy consumption that may be introduced by the particular MAC protocol (for instance, $E_{l}^{DAR}$ in TinyOS).

In the case of LPP-based protocols, where typically a probe packet is repetitively transmitted by the node acting as receiver, the formulation is slightly different:

(8) $$\varepsilon \left[E_{round}\left(X\right)\right]≅\sigma \left(X\right)\cdot E_{rxpkt}+\left(\sigma \left(X\right)+1\right)\cdot E_{txpkt}\;+E_{l}\frac{T_{rnd}}{T_{l}}\frac{DC\left(X\right)}{100}+\sigma \left(X\right)\cdot E_{trigger}\left(X\right),$$

(9) $$E_{trigger}\left(X\right)=\overset{CH\left(X\right)}{\underset{i=1}{\sum }}\frac{1+\sigma \left(c_{i}\left(X\right)\right)}{\sigma \left(X\right)}\cdot E_{trigger,i}\left(X\right),\;=\overset{CH\left(X\right)}{\underset{i=1}{\sum }}\frac{1+\sigma \left(c_{i}\left(X\right)\right)}{\sigma \left(X\right)}\cdot (\varepsilon_{i}\left[n\right]E_{txprobe}+E_{extra}).$$

The term $c_{i}\left(X\right)$ represents a child node of node X, with i varying between 1 and CH(X), the total number of children of node X, and $\varepsilon_{i}\left[n\right]$ is the expected number of transmissions of the probe packet from node X to node $c_{i}\left(X\right)$. The term $E_{trigger,i}\left(X\right)$ denotes the energy wasted by node X to trigger the communication of every packet from its child node $c_{i}\left(X\right)$. Also note that $\sigma \left(X\right)=\sum_{i=1}^{CH\left(X\right)}\left(1+\sigma \left(c_{i}\left(X\right)\right)\right)$, with $\sigma \left(c_{i}\left(X\right)\right)$ the traffic load of node $c_{i}\left(X\right)$. Hence, Equation (9) can be viewed as a weighted average.

Equations (5) and (8) can be assimilated into the following equation, since the reporting time is always much larger than the duration of duty periods ($\frac{T_{rnd}}{T_{l}}\gg 1$):

(10) $$\varepsilon \left[E_{round}\left(X\right)\right]≅\sigma \left(X\right)\cdot (E_{rxpkt}+E_{txpkt}+E_{trigger}\left(X\right))\;+E_{l}\frac{T_{rnd}}{T_{l}}\frac{DC\left(X\right)}{100}.$$

In summary, Equation (10) characterizes, in an approximate way, the energy consumption (per round) of nodes in time-driven WSN implementing LPL-based or LPP-based duty-cycled MAC protocols. Whereas the specificity of the MAC protocol is embedded into the term $E_{trigger}\left(X\right)$, the important fact regarding the subsequent analysis is the dependence of the energy consumption per round on the traffic load, represented by σ(X). Next, based on Equation (10), the condition for energy neutral operation is formulated.

## 3. Solar Energy-Harvesting Models

The literature on solar-based energy harvesting models is extensive. A good classification is provided in [23]. Here, a first distinction is established between models for the solar irradiance (environmental models), and models for the solar energy-harvesting devices (harvester models). Harvester models are typically used for design or energy management optimization purposes. In turn, they can be classified into high-level and low-level. High-level models ignore the details about the actual hardware implementation of the harvester; rather, the harvester is viewed as a black-box that performs an energy transfer function between the actual solar radiation and the energy that feeds the sensor node. Accordingly, efficiency is the magnitude that best characterizes the performance of this energy transfer process. Since high-level models are relatively simple, their analytical formulation is feasible [24,25] and can be easily integrated into general-purpose simulation software environments. In contrast, low-level models are highly implementation-dependent and hence tightly associated with specific architectures. Low-level modeling relies on equivalent circuits of the integrated components, which are then combined into a system model to accurately describe the behavior of a given hardware architecture [26,27,28]. These low-level models can be implemented via specialized simulation software environments, like SPICE [29] or Simulink (a visual programming tool for model-based design that supports automatic code generation in Matlab), or directly executed by using EH-WSN-oriented simulation tools, like GreenCastalia [30], SolarCastalia [31], SensEH [32] and others, which already include an energy module that integrates the energy-harvesting, rechargeable battery and energy consumption models.

Environmental models focus on the estimation of solar irradiance. As stated in [23], they can be further classified into statistical models and astronomical models. Statistical models make use of historic measurements of solar irradiance to predict future values of this magnitude at locations close or similar to the measurement points [33,34,35,36,37]. Optimized versions of the predictive algorithms used for these purposes can also be incorporated into node operation in order to obtain real-time estimates of future solar irradiance and correspondingly adjust the node duty cycle [38]. Irradiance measurements are typically stored in large databases, like Meteonorm [39] or the NASA POWER Project database [40]. Since these databases are publicly available, the collected data can also be used to conduct simulations based on real radiation levels [41]. In contrast to statistical models, astronomical models calculate solar irradiance on the basis of geometric calculations that include the solar panel inclination, the panel orientation, the latitude, the day of the year and the time of the day. So, in essence, these are deterministic models that do not take into account weather conditions or local obstacles. Some examples can be found in [42,43]. These models are relatively simple and thus they can be easily included in analytical methods. Since the focus of the present paper is not on the characterization of solar irradiance, but on the determination of the optimal routing strategy for EH-WSN and the comparison of this strategy with other alternatives, any model can be used as long as all alternatives are compared under the same conditions. Consequently, a simple analytically-tractable model is preferable. In particular, the model proposed in [44] has been used for numerical evaluation (Section 6). This model, in addition to formulating solar irradiance as a simple quadratic function of the time of the day (for given parameters about location and month of the year), is consistent with the accurate model proposed in [43].

## 4. Energy Neutral Operation

In contrast to conventional battery-powered WSN, which are designed with the objective of maximizing network lifetime, in the case of EH-WSN, the objective is to maximize performance under self-sustained operation. More formally, this condition is known as Energy Neutral Operation (ENO), which essentially means that, in a given period of time, the energy balance at a node is non-negative. Also, many energy-harvesting mechanisms in sensor networks obey the so-called harvest-store-consume supply alternative, which consists of combining the energy-harvesting subsystem with a buffer for energy storage (rechargeable battery or supercapacitor) [3]. According to this model, and assuming that the energy buffer does not have any inefficiency in charging and does not leak any energy over time, ENO can be mathematically formulated as follows [45] (the notation has been adapted):

(11) $$E\left(t\right)=E\left(0\right)+\int_{0}^{t}P_{out}\left(u\right)du-\int_{0}^{t}P_{c}\left(u\right)du\geq 0,\;\forall t\geq 0.$$

In this expression, E(t) denotes the energy balance at time t, $P_{out}\left(t\right)$ is the output power delivered by the harvesting subsystem at time t, $P_{c}\left(t\right)$ is the power consumed by the device at time t and, obviously, E(0) is the energy initially stored in the buffer. Let us assume that the energy source exhibits a regular pattern, with periodicity $T_{S}$ (energy-harvesting period). Accordingly, ENO can be formulated for one period $T_{S}$, since this is typically a very large multiple integers of the reporting period given by $T_{rnd}$. For instance, the most representative periodic source is the sunlight (really, it is quasi-periodic, but this will be considered further), as photovoltaic circuits constitute the most efficient form of energy conversion, at least for current sensor networks. In this case, $T_{S}$ corresponds to a one-day interval, which is much larger than usual reporting periods (one or several minutes). For the same reason, we can undoubtedly assume that the energy consumed by the sensor node is uniformly distributed over the round duration, implying that power consumption is independent of time: $P_{c}\left(t\right)=P_{c}=\frac{E_{round}}{T_{rnd}}$. Under these assumptions, ENO can be reformulated for a given node X by imposing that the energy at the beginning of an energy-harvesting period is equal to the energy at the beginning of the previous energy-harvesting period:

(12) $$E\left(T_{S},X\right)=E\left(0,X\right)+\int_{0}^{T_{S}}P_{out}\left(u,X\right)du-\int_{0}^{T_{S}}\frac{E_{round}\left(X\right)}{T_{rnd}}du=E\left(0,X\right).$$

Note that, in order to guarantee that E(t,X)≥0 ∀t, a condition on E(0,X) (initial energy) must also be fulfilled. Moreover, sufficiently large values of E(0,X) release nodes from the need to enter a recharging state, even if significant irregularities occur during the energy-harvesting process (for instance, cloudy days in the case of solar-based sensor networks). However, despite the importance of E(0,X) as an energy repository that mitigates the irregularities of the energy sources considered in this paper, its mathematical formulation has been omitted here as it is not relevant to the subsequent analysis. Then, by combining Equations (10) and (12), we can end up with the following expression for the duty cycle of node X in terms of its energy harvesting capability and traffic load:

(13) $$\frac{DC\left(X\right)}{100}≅\frac{E_{out}\left(T_{S},X\right)}{E_{l}}\frac{T_{l}}{T_{S}}\;-\sigma \left(X\right)\cdot \frac{E_{rxpkt}+E_{txpkt}+E_{trigger}\left(X\right)}{E_{l}}\frac{T_{l}}{T_{rnd}}.$$

Here, $E_{out}\left(T_{S},X\right)=\int_{0}^{T_{S}}P_{out}\left(u,X\right)du$. Equation (13) extends the ENO condition obtained in [14] to regular energy sources and generic duty-cycled MAC protocols. It makes it explicit the dependence of the duty cycle on the specific operating conditions of each node in the network, namely energy harvesting capability and traffic load. In particular, the presence of the energy harvesting term contributes to achieving much larger duty cycles in EH-WSN than those obtained in battery-powered WSN. Besides, Equation (13) reveals that the duty cycle decreases as the traffic load increases.

## 5. Criterion for Optimal Routing

It is well known that, in EH-WSN, enhancing performance under self-sustained operation implies maximizing the duty cycle of nodes as much as possible. According to expression (13), maximizing the duty cycle of any node requires minimizing its traffic load (for the rest of the parameters remaining fixed). However, reducing the traffic load of a node may be achieved at the expense of increasing the traffic load of nearby nodes. So, the emphasis will be put on the average traffic load across the network. In a global sense, minimizing the average traffic load across the sensor network will contribute to maximizing the average duty cycle of nodes, fact that in turn will contribute to improving performance metrics.

**Figure 4:**
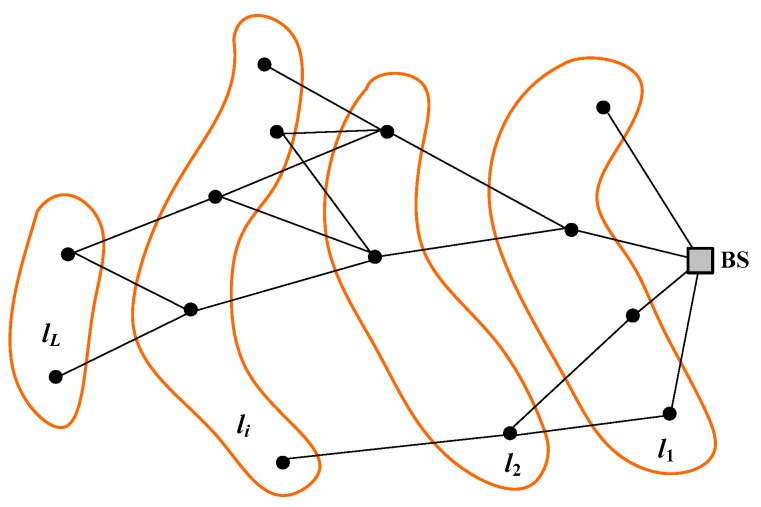
Layer decomposition of a connected network. Lines represent feasible links for the given transmission range. Only inter-layer links are drawn. BS denotes the base station.

The problem of minimizing the average traffic load can be addressed by decomposing the sensor network into layers. Let us assume that the transmission range of all nodes is r, and let us define $l_{1}$ as the subset of nodes that are at a distance not greater than r from the base station. Next, let us define $l_{2}$ as the subset of nodes that are in the transmission range of at least one node in $l_{1}$ but at a distance greater than r from the base station, $l_{3}$ as the subset of nodes that are in the transmission range of at least one node in $l_{2}$ but out of the transmission range of all nodes in $l_{1}$, and so on. In this layer decomposition process, it is assumed that a layer exists if it contains at least one element (node), and that the existence of layer $l_{i}$ implies the existence of layer $l_{i-1}$, for any i=2…L. Figure 4 shows an example of layer decomposition for a connected network. Note that, if N is the number of sensor nodes in the network and L is the number of (non-empty) layers, the following properties hold:

- L≤N. The equality corresponds to the case where each layer contains a single node.
- $l_{i}\cap l_{j}=\emptyset ,\;\forall i\neq j$. This property is a direct consequence of the above definition of layer.
- If the network is connected, $l_{1}\cup l_{2}\cup \ldots \cup l_{L}=u$, where u represents the set of all sensor nodes.
- If the network is disconnected, $l_{1}\cup l_{2}\cup \ldots \cup l_{L}\subset u$.

Once it has been verified that the network is connected, the next step is to find an appropriate routing topology. In our context, this means determining a spanning tree rooted at the base station that minimizes the average traffic load across the network. To achieve this goal, let us first define $n_{i}$, with i=1…L, as the size of layer $l_{i}$, that is, the number of nodes contained in this layer. Obviously, $\sum_{i=1}^{L}n_{i}=N$. If we assume, with no loss of generality, that each node sends one packet per reporting period, then the traffic load supported by node $x_{k}$, namely $\sigma \left(x_{k}\right),\;k=1\ldots N$, is the total number of descendants of this node in the spanning tree. Let us also assume that only inter-layer connections (directed towards the base station) are allowed. In this case, the following lemma holds:

**Lemma** **1.** *If*$\bar{\sigma }$*denotes the average traffic load supported by a network that only contains inter-layer connections, the following equation holds:*$\bar{\sigma }=\frac{1}{N}\sum_{i=1}^{L-1}\sum_{j=i+1}^{L}n_{j}$*, with*$n_{j}$*the size of layer*$l_{j},\;j=1\ldots L$.

**Proof.**  Let $\bar{\sigma_{i}}$ be the average traffic load supported by nodes in layer $l_{i},\;i=1\ldots L$. Then, $\bar{\sigma_{L}}=0$ and $\bar{\sigma_{i}}$ = $\frac{\sum_{j=i+1}^{L}n_{j}}{n_{i}},\;i=1\ldots L-1$. The first part of the statement is obvious, since $\sigma \left(x_{k}\right)=0\;\forall x_{k}\in l_{L}$ (nodes in the last layer do not receive packets from other nodes). For the second part, let us first consider layer $l_{L-1}$. In this case, regardless of the specific inter-layer connections between this layer and layer $l_{L}$, we have $\sum_{x_{k}\in l_{L-1}}\sigma \left(x_{k}\right)=n_{L}$: Since all input links to nodes in layer $l_{L-1}$ come from nodes in layer $l_{L}$, and each node generates one packet per reporting period, the overall traffic load carried out by layer $l_{L-1}$ coincides with the number of nodes in layer $l_{L}$. Accordingly, the average traffic load supported by layer $l_{L-1}$ is $\bar{\sigma_{L-1}}=\frac{\sum_{x_{k}\in l_{L-1}}\sigma \left(x_{k}\right)}{n_{L-1}}=\frac{n_{L}}{n_{L-1}}$. Next, since all input links to nodes in layer $l_{L-2}$ come from nodes in layer $l_{L-1}$, we can state that $\sum_{x_{k}\in l_{L-2}}\sigma \left(x_{k}\right)=\sum_{x_{k}\in l_{L-1}}\left(1+\sigma \left(x_{k}\right)\right)$, because each node $x_{k}\in l_{L-1}$ generates one packet and forwards a number of packets equal to its traffic load. Moreover, we can state that $\sum_{x_{k}\in l_{L-1}}\left(1+\sigma \left(x_{k}\right)\right)=n_{L-1}+\sum_{x_{k}\in l_{L-1}}\sigma \left(x_{k}\right)=n_{L-1}+n_{L}$, which means that, with only inter-layer connections, the traffic load supported by layer $l_{L-2}$ is equal to the total number of nodes in layers $l_{L-1}$ and $l_{L}$. In turn, this implies that the average traffic load supported by nodes in layer $l_{L-2}$ is given by $\bar{\sigma_{L-2}}=\frac{n_{L-1}+n_{L}}{n_{L-2}}$. So, from the point of view of layer $l_{L-2}$, all nodes in layers $l_{L-1}$ and $l_{L}$ can be grouped into a single $l_{L-1}\cup l_{L}$ super-layer. Then, by iterating this procedure over subsequent layers, we can end up with a general expression for the average traffic load supported by any layer: $\bar{\sigma_{i}}=\frac{\sum_{j=i+1}^{L}n_{j}}{n_{i}},\;i=1\ldots L-1$. Finally, the average traffic load supported by the entire network can be expressed as $\bar{\sigma }=\sum_{i=1}^{L}\bar{\sigma_{i}}\cdot \frac{n_{i}}{N}=\frac{1}{N}\sum_{i=1}^{L-1}\sum_{j=i+1}^{L}n_{j}$. □

The following lemma demonstrates that a routing tree based exclusively on inter-layer connections minimizes the average traffic load:

**Lemma** **2.** *Let*${\bar{\sigma }}^{\left(c\right)}$*the average traffic load that results from applying a given routing criterion*c*to construct the spanning tree. If*${\bar{\sigma }}^{*}$denotes the average traffic load obtained by applying the “only inter-layer connections” criterion, the following statement is true:${\bar{\sigma }}^{*}=\underset{c\in C}{\min}\;\left\{{\bar{\sigma }}^{\left(c\right)}\right\}$*, with*C*the set of all possible criteria.*

**Figure 5:**
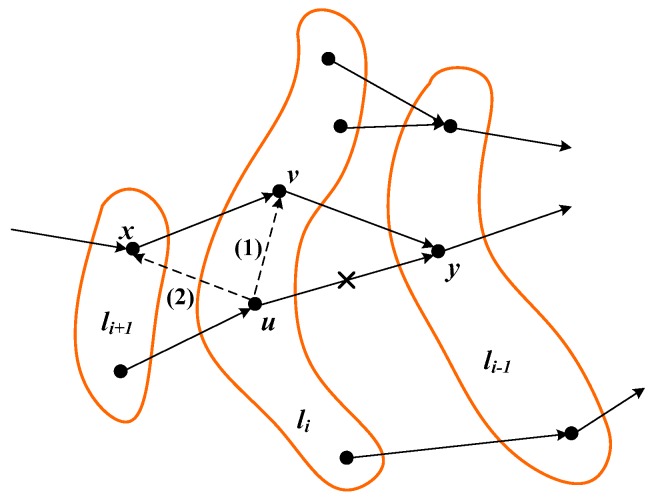
Converting an inter-layer connection into an intra-layer (1) or a backward inter-layer (2) connection.

**Proof.**  In general, any routing criterion different from “only inter-layer connections” will give rise to at least L−1 inter-layer connections (as there must be at least 1 inter-layer connection between two successive layers) combined with several intra-layer and/or backward inter-layer connections (see Figure 5). Let us first focus only on intra-layer connections. Figure 5 shows the simplest variation that can be introduced into a spanning tree that initially contains inter-layer connections exclusively. As it can be noticed, node u belonging to layer $l_{i}$ is reconnected to node v in the same layer. The new connection, labeled (1), only causes an increase in the traffic load supported by node v. In effect, if $\sigma_{old}\left(x_{k}\right)$ and $\sigma_{new}\left(x_{k}\right)$ denote, respectively, the traffic load of any given node $x_{k}$ before and after the reconnection, we have:

- $\sigma_{new}\left(u\right)=\sigma_{old}\left(u\right)=\sigma \left(u\right)$.
- $\sigma_{new}\left(v\right)=\sigma_{old}\left(v\right)+1+\sigma \left(u\right)$, with $\sigma_{old}\left(v\right)=1+\sigma \left(x\right)$.
- $\sigma_{old}\left(y\right)=1+\sigma_{old}\left(v\right)+1+\sigma \left(u\right)=3+\sigma \left(x\right)+\sigma \left(u\right)$.
- $\sigma_{new}\left(y\right)=1+\sigma_{new}\left(v\right)=1+\sigma_{old}\left(v\right)+1+\sigma \left(u\right)=3+\sigma \left(x\right)+\sigma \left(u\right)=\sigma_{old}\left(y\right)$.

So, the reconnection only increases the traffic load of node v, whereas the traffic load of the rest of the nodes remain unchanged. Accordingly, the average traffic load of the layer containing node v increases, whereas the average traffic load of the rest of layers does not experience any change. Altogether, this means that the average traffic load calculated over the entire network increases for routing criteria that generate intra-layer connections.

Let us focus now on the effects of backward inter-layer connections. Figure 5 shows an elementary change, where a (forward) inter-layer connection from node u to node y is replaced by a backward inter-layer connection to node x. The new balance is as follows:

- $\sigma_{new}\left(u\right)=\sigma_{old}\left(u\right)=\sigma \left(u\right)$.
- $\sigma_{new}\left(x\right)=\sigma_{old}\left(x\right)+1+\sigma \left(u\right)$
- $\sigma_{new}\left(v\right)=1+\sigma_{new}\left(x\right)=1+\sigma_{old}\left(x\right)+1+\sigma \left(u\right)=\sigma_{old}\left(v\right)+1+\sigma \left(u\right)$.
- $\sigma_{old}\left(y\right)=1+\sigma_{old}\left(v\right)+1+\sigma \left(u\right)$.
- $\sigma_{new}\left(y\right)=1+\sigma_{new}\left(v\right)=1+\sigma_{old}\left(v\right)+1+\sigma \left(u\right)=\sigma_{old}\left(y\right)$.

So, now the reconnection causes an increase in the traffic load of nodes x and v, whereas the traffic load of the rest of the nodes remain unchanged. Accordingly, only the average traffic load supported by layers $l_{i+1}$ and $l_{i}$ increases, meaning that the average traffic load calculated over the entire network increases for routing criteria that generate backward inter-layer connections. In summary, any routing scheme generating intra-layer and/or backward inter-layer connections will incur an average traffic load larger than the average traffic load produced by a routing scheme that only generates (forward) inter-layer connections. □

Note that creating a routing topology that only includes (forward) inter-layer connections is equivalent to applying the minimum hop count criterion. Therefore, this is the optimal routing strategy for time-driven duty-cycled EH-WSN under regular energy sources. 

## 6. Numerical Results

In order to validate the theoretical results and demonstrate the impact of the routing strategy on the average duty cycle of nodes, several simulation experiments were conducted by varying the network size from 100 to 1000 sensor nodes (in steps of 100). The data, shown in Table 1, which correspond to TinyOS sensor nodes, were used. Correspondingly, the transmission range (r) was set to 250 m. The sensor field consisted of a square region of 1 km^2^, with the left lower corner and the base station respectively located at coordinates (0, 0) and (1000, 500) (in meters). As for the regular energy source, solar radiation was considered. As stated in Section 3, the solar energy-harvesting model proposed in [44] was used, with the meteorological data taken from the NASA POWER Project database for the city of Madrid and the month of September: $D_{month}=4.87$ kWh/m^2^/day and STDHOURS=12.5 hours [40]. Three routing protocols were considered in the evaluation: A routing protocol based on the MHC criterion, the well-known Collection Tree Protocol (CTP) and a generic location-based or Geographical Routing Protocol (GRP). CTP has been adopted in this evaluation because of its extended use in both, battery-powered and energy-harvesting wireless sensor networks. An example is the case of TinyOS-based sensor networks, where CTP runs on top of a duty-cycled LPL-based MAC protocol. To be more precise, La-CTP should be considered, but since a duty-cycled LPL protocol was supposed to be running at the MAC layer, loops could not happen and, consequently, it could be expected that La-CTP provided the same results as CTP. In CTP, the expected number of transmissions (ETX) is adopted as the routing metric, and then Shortest Path Routing (SPR) is applied to determine the least cost path from every node to the base station [46]. Note that the number of transmission tries is a magnitude that depends on both the quality of the link that connects the two nodes and the asynchrony between their duty periods (recall Figure 1). Since the purpose of this simulation is to compare the intrinsic effects of MHC, CTP and GRP on the average traffic workload, it has been assumed that all feasible links are in good quality conditions. Accordingly, the only factor determining the number of transmission tries is the asynchrony between duty periods. As stated in [14], the number of transmission tries between a transmitter and a receiver (parent) node is a fixed value extracted from a quasi-uniform distribution between 1 and a maximum value that depends on the duty cycle of the receiver (parent) node. Note that this introduces a “snake biting its tail” problem when dealing with CTP routing in the context of EH-WSN, in which the duty cycle varies from node to node: The number of tries required by a given node depends on the duty cycle of its parent node, which in turn depends on the routing topology created by CTP based on the number of tries. In fact, this is a typical behavior observed in CTP: It enters an initial transient period during which a connected network is progressively built, and after that the routing topology becomes practically fixed, consistently with the regular traffic conditions imposed by time-driven applications. In such a steady-state regime, the traffic load and duty cycle of each node becomes stable, at least during long periods of time. Based on the data shown in Table 1, the simulation experiments performed in [14] and the large network densities managed in the current simulation (note that a large network density means a large number of nodes distributed over a relatively small number of layers), a uniform distribution between 1 and 10 becomes sufficiently representative to characterize the number of tries throughout all feasible links in the network. Finally, another important category of routing protocols for sensor networks encompasses those based on location information [47,48]. Though these protocols were developed for battery-powered WSN, they could be perfectly extended to EH-WSN. In essence, these protocols take advantage of location information to make routing more efficient. As indicated in [47], either real or virtual geographical coordinates can be used (in the first case, sensor nodes are assumed to be equipped with a Global Positioning System or GPS). Protocols in this category use location information in multiple forms, depending on how a node holding a packet selects the next-hop node in the route towards the destination (base station). The neighbor which is closest (in terms of Euclidean distance) to the destination, the most distant neighbor that is closer to the destination (most-forward-within-radius technique), the nearest neighbor that is closer to the destination (nearest-forward-process) or the neighbor with the minimum angular distance from the imaginary line that connects the current node to the destination (compass routing), are just some examples. A common feature of these variations is that they introduce intra-layer connections that increase the average traffic workload over the network. Since this is the relevant fact in the present evaluation, a generic geographical routing protocol under the name of GRP has been simulated, leaving aside the peculiarities of each variation. Specifically, in GRP the next-hop node is randomly selected among all feasible intra-layer and forward inter-layer connections of the current node.

**Figure 6:**
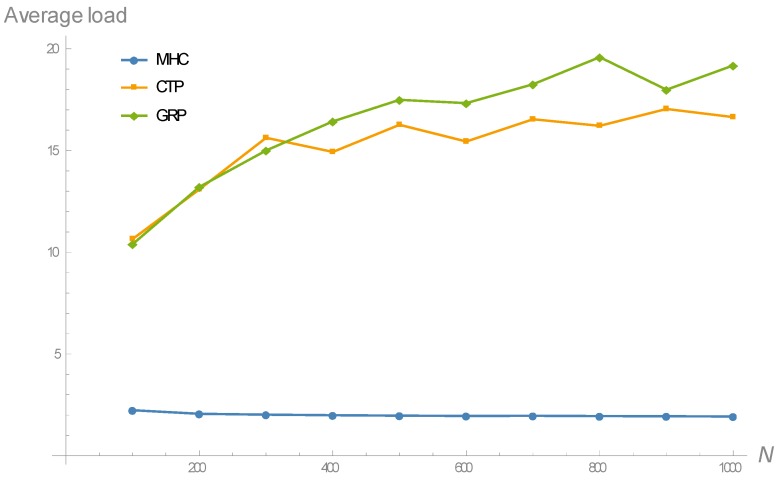
Evolution of the average traffic load with regard to the network size, for the routing metrics considered in the analysis. The surprisingly small value obtained for MHC is a consequence of the large network densities managed in the simulation.

For each network size and routing protocol, the simulation experiment consisted of 30 simulation runs, each providing two results: The average traffic load and the average duty cycle over the network. Particularly, Figure 6 shows the evolution of the average traffic load as the network size increases, for the three routing schemes considered in the analysis. As it can be noticed, MHC leads to significantly lower traffic load per node compared to the rest of routing criteria; this is because the latter generates an intra-layer, in addition to forward inter-layer connections, whereas the former only gives rise to forward inter-layer connections. Moreover, MHC also exhibits a practically flat behavior of around two forwarded packets per node on average, meaning that it is the routing metric that best distributes the overall traffic load across the network. Therefore, MHC is substantially more scalable in terms of network size than the rest of criteria.

**Figure 7:**
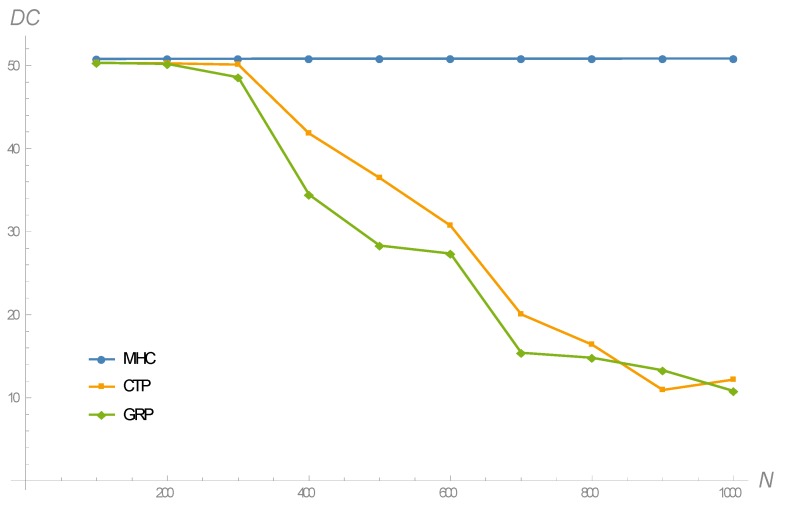
Evolution of the average duty cycle in terms of the network size, for different routing criteria.

The results in terms of the duty cycle of nodes are shown in Figure 7. This figure is a direct consequence of Figure 6 and Equation (13). Leaving aside the energy-harvesting term, Equation (13) defines the duty cycle of node X as a function of (1) the duty cycle of its parent node, through the term $E_{trigger}\left(X\right)$, and (2) the number of descendant nodes, through the term σ(X). Accordingly, Algorithm 1 outlines the main steps to calculate the duty cycle of every node in the network. As it can be noticed, this algorithm proceeds by layers, though the concept of layer has been slightly modified here, since it is now based on the spanning tree that results from applying any routing protocol. In other words, for any given spanning tree, the first layer is constituted by the nodes directly connected to the base station; the second layer contains the nodes directly connected to the nodes in the first layer; the third layer groups the nodes directly connected to the nodes in the second layer; etc. So, for instance, a node in the second layer could belong to the first layer according to the concept of layer adopted in Section 4. To conclude the description of Algorithm 1, its computational complexity can be evaluated. Given that the network size is N, it can be shown that the algorithm requires $N\left(N+1\right)/2\sim N^{2}$ matrix explorations (assuming that the spanning tree is given in matrix form), N sums and N evaluations of the duty cycle according to Equation (13).


**Algorithm 1: Evaluation of the duty cycle**
Let S be a symmetric (N+1)x(N+1) matrix representing the given spanning tree.Let $S_{ij}$ be an element of the matrix, with i,j=0…N (node 0 represents the base station).Let $S_{ij}=1$ if there is a direct connection between nodes i and j, and $S_{ij}=0$ otherwise.By inspecting element by element of the upper (lower) triangle of matrix S, determine the set of layers $SL=\left\{layer_{i},i=1\ldots NL\right\}$, with NL the number of layers, and the set of children of every node $x_{k}\in layer_{i}$. Note that $layer_{i}\cap layer_{j}=\emptyset \;\forall i\neq j$ and $layer_{1}\cup \ldots \cup layer_{NL}=U$, with U the whole set of sensor nodes in the network (universal set).
**for**
i=NL
**downto**
i=1
**do**
  Calculate $\sigma \left(x_{k}\right)$ for every node $x_{k}\in layer_{i}$. Here, recall the general expression provided in Section 2: $\sigma \left(X\right)=\sum_{i=1}^{CH\left(X\right)}\left(1+\sigma \left(c_{i}\left(X\right)\right)\right)$. Note also that $\sigma \left(x_{k}\right)=0\;\forall x_{k}\in layer_{NL}$.
**for**
i=1
**to**
i=NL
**do**
  Obtain $DC\left(x_{k}\right)$ for every node $x_{k}\in layer_{i}$, by taking into account that the duty cycle of the base station is 100%.  **if**
$DC\left(p(x_{k})\right)==0$
**then**
$DC\left(x_{k}\right)=0$  **else**    Calculate $DC\left(x_{k}\right)$.    **if**
$DC\left(x_{k}\right)<0$
**then**
$DC\left(x_{k}\right)=0$Calculate the average duty cycle: $DC=\frac{1}{N}\sum_{x_{k}\in U}DC\left(x_{k}\right)$.

As it can be noticed from Figure 7, all routing criteria give rise to very similar duty cycles (around 50%) for moderately large network sizes of up to 300 nodes approximately. However, as the network size increases beyond this value, only MHC is capable of maintaining such a 50% duty cycle, whereas CTP and GRP exhibit highly-decreasing trends. Note that the gap between MHC and CTP might appear to be surprising, since CTP searches to minimize the link-level delay, which is also a consequence of the duty cycle maximization pursued by MHC. However, there is a subtle difference that explains this gap: CTP decides the next-hop neighbor based on the immediate number of transmission tries, a fact that leads it to select intra-layer in addition to forward inter-layer connections. This behavior increases as the network size increases, and hence the decreasing trend, as shown in Figure 7. On the other hand, a small duty cycle has a negative impact on the whole distribution of the number of transmission tries, whose upper bound increases. So, indeed CTP selects the shortest routes based on the link-level delays, but these result from increasingly spread out distributions. In contrast, MHC selects exclusively forward inter-layer connections, which allows nodes to sustain large duty cycles even under increasing network sizes. Consequently, link-level delays in MHC-based routing obey distributions with smaller variances than those corresponding to CTP.

## 7. Conclusions

In this paper, first a generic model has been developed to relate the duty cycle and traffic load of any node in a time-driven duty-cycled EH-WSN. This model results from a relatively simple extension of a previous result on the energy consumed by TinyOS nodes executing time-driven applications. Then, the focus has been put on the routing strategy. Specifically, it has been demonstrated that the MHC criterion minimizes the average traffic load across the network and maximizes the average duty cycle of nodes. Note that this is a primary goal in energy-harvesting WSN, since larger duty cycles are expected to optimize network performance.

The main result obtained in this paper has been validated via simulation by comparing MHC with other relevant routing protocols, such as CTP and a generic GRP (though any protocol that generated intra-layer connections would have served the same purposes). Thus, this comparison encompasses three widespread and at the same time quite different routing criteria for sensor networks: Number of hops, number of transmission tries and geographical distance. Simulations results reveal that MHC substantially outperforms the other protocols, especially beyond moderately large network sizes. This also represents a better performance in terms of scalability. Accordingly, this paper suggests assigning top priority to the MHC criterion in the development of routing protocols for time-driven duty-cycled EH-WSN.

An important issue for further research in EH-WSN is to determine the optimal routing strategy when other conditions are taken into account: Power control enabled, transmission impairments, locally poor energy-harvesting situations and even non-regular energy sources. Also, a predictive algorithm, like any of those referred to in Section 3, could be included into node operation in combination with the routing protocol. In this way, the node would be able to obtain estimates of future energy intakes and dynamically adjust its duty cycle and routing topology if necessary.

## Figures and Tables

**Table 1:** Parameters used in the validation of the approximate energy consumption model for LPL TinyOS nodes. RX and TX denote, respectively, the reception and transmission modes of operation.

Magnitude	Value
Bandwidth	250 Kbps
$T_{pkt}$	1.312 ms
$T_{ack}$	0.544 ms
$T_{CCA}$	0.4 ms
$W_{ack}$	1 ms
$T_{l}$	5 ms
DAR	100 ms
$T_{rnd}$	60 s
Voltage	3 V
Current draw in RX	18.8 mA
Current draw in TX (at 0 dBm)	17.4 mA
